# Edible Insects and Global Food Security

**DOI:** 10.3390/insects12050472

**Published:** 2021-05-19

**Authors:** Fabio Verneau, Mario Amato, Francesco La Barbera

**Affiliations:** Department of Political Science, University of Naples Federico II, Via Rodinò 22/A, 80138 Naples, Italy; verneau@unina.it (F.V.); francesco.labarbera@unina.it (F.L.B.)

Starting in 2008 and lasting up until 2011, the crisis in agricultural and, in particular, cereal prices triggered a period of riots that spread from the Mediterranean basin to the rest of the world, reaching from Asia to Central America and the African continent. The 2008–2011 crisis and related food riots re-proposed global food security as one of the most important issues on the political agendas of many countries and international organizations.

According to the most recognized definition established by the World Food Summit in 1996, food security is a ‘‘situation that exists when all people, at all times, have physical, social, and economic access to sufficient, safe, and nutritious food that meets their dietary needs and food preference for an active and healthy life’’. The definition is based on four pillars: availability, stability, access and utilization. The first dimension relates to the availability of sufficient food, and therefore, it refers to the overall ability of the agricultural system to meet food demand. The stability dimension relates to individuals who are at high risk of temporarily or permanently losing their access to the resources needed to consume adequate food. The third dimension, access, covers access by individuals to adequate resources to acquire appropriate foods for a nutritious diet. Thus, a key element is the purchasing power of consumers and the evolution of real incomes and food prices. Finally, utilization encompasses all food safety and quality aspects of nutrition.

Beyond the food crisis that relaunched the issue of global food security, many factors may aggravate the balance between global food supply and demand in the coming decades, thus affecting the level of food insecurity.

Population growth, urbanization, dietary demand and Westernization of food styles in the Far East, overexploitation and depletion of natural resources, climate change and land use for biofuel production are just some of the crucial factors that may jeopardize food security goals in the coming years.

In particular, the dynamics of growth in the global demand for proteins, and especially animal proteins, have directed the attention of researchers to the identification of new protein sources that can meet the growing global demand.

Categories of the alternative sources of animal proteins include: cultured meats, produced in vitro; plant-based meat analogs, manufactured using plants-extracted proteins; single-cell proteins (SCP), characterized by a microbial origin; earthworms and edible insects, which exhibit a high feed/meat conversion rate.

Among the alternative sources of animal proteins mentioned so far, edible insects represent the option that most closely meets the necessary requirements for food security.

As regards the availability pillar, edible insects would provide notable quantities of animal proteins while relieving pressure on the allocation of land for livestock production. In addition, edible insects could satisfy the stability and access pillars since small farms could be established to support local rural communities, mitigating periods of scarcity while providing a good level of access to food. Lastly, both under the aspects of safety and quality that can be linked to the fourth pillar of utilization, edible insects represent a very promising option thanks to their high nutritional profile and a high level of food safety, as has been recently pointed out by the European Food Safety Agency (EFSA).

Hence, it is not surprising to observe the rising interest of scholars in relation to entomophagy (eating insects) and indirect entomophagy (eating animals fed with insects) across different disciplinary perspectives; in the current special issue, several timely topics are investigated by the contributors. Tedesco and colleagues [1] contribute to the risk assessment of insect-based food, which is key for consumers’ health and safety; they also provide empirical evidence of critical aspects and possible resolutions. In addition, the authors explore the connection between entomophagy, food rearing and waste reduction, which is very important in relation to the sustainability of insect-based food.

Beside the fundamental aspects connected to food safety and consumers’ health, recent research has shown the importance of understanding the attitudes and beliefs that determine the willingness to eat insects, directly and indirectly. In this research path, Fasanelli and colleagues [2] study—for the first time in contemporary research—the social representations that different categories of consumers have developed and hold as regards entomophagy. Using a mixed-methods approach, the authors contribute to a better understanding of consumers’ beliefs, their structure and relative importance, thus allowing insight into communication strategies. The topic of willingness to eat insects is further explored by Moruzzo and colleagues [3], who confirm the relevance of food neophobia in orienting consumers’ preferences. In this respect, the role of information has been investigated by Menozzi and colleagues [4], who highlighted that providing information on sustainability and nutritional benefits increases consumers’ knowledge, and consequently, reduces their aversion towards using insects as a livestock feed source.

As we mentioned, recent literature has repeatedly shown the benefit of adding insect-based foods to the human diet. Nevertheless, several topics remain underexplored. First, while the benefits of using insects for food and livestock feed are clear, the possibility of large-scale production and commercialization, and the probability of spreading insects as food and feed in everyday life, are still unclear. Despite the huge literature on consumer acceptance of insect-based food, segmentation of this possible future market has never been proposed. Verneau and colleagues [5] try to fill this gap with market segmentation based on the Food-Related Lifestyle Scale [6]. Second, the main part of this research on consumers’ attitudes and beliefs about direct and indirect entomophagy has been carried out in developed countries, mostly in Europe, whereas less research has been conducted in less developed countries. In this regard, the efforts of Hlongwane and colleagues [7], who studied knowledge and beliefs in an indigenous context in South Africa, remarkably expand our scientific understanding of these topics outside of the most investigated and proximal research contexts. Third, research investigating consumers’ attitudes towards direct and indirect entomophagy has been somewhat hindered by the absence of specific and validated measures. Recently, La Barbera and colleagues developed the Entomophagy Attitude Questionnaire (EAQ), which has been validated in several languages and contexts and shows excellent psychometric proprieties [8]. In the current special issue, La Barbera and colleagues provide a further exploration of the nomological validity of the EAQ, considering the perceived risk of a sample of consumers as regards eating raw insects and different animals fed with insects [9].

Hence, the contributions collected by this special issue explore current questions and offer relevant answers, while demonstrating the vibrancy of research on edible insects and global food security.
